# Continuous use of glycomacropeptide in the nutritional management of patients with phenylketonuria: a clinical perspective

**DOI:** 10.1186/s13023-021-01721-8

**Published:** 2021-02-13

**Authors:** Maria João Pena, Alex Pinto, Manuela Ferreira de Almeida, Catarina de Sousa Barbosa, Paula Cristina Ramos, Sara Rocha, Arlindo Guimas, Rosa Ribeiro, Esmeralda Martins, Anabela Bandeira, Cláudia Camila Dias, Anita MacDonald, Nuno Borges, Júlio César Rocha

**Affiliations:** 1 Departamento de Biomedicina, Unidade de Bioquímica, Faculdade de Medicina, Universidade do Porto, 4200-319 Porto, Portugal; 2 Department of Dietetics, Birmingham Children’s Hospital, Birmingham, B4 6NH UK; 3 Faculty of Health and Human Sciences, University of Plymouth, Plymouth, PL6 8BH UK; 4 Centro de Genética Médica, Centro Hospitalar Universitário Do Porto (CHUP), 4099-028 Porto, Portugal; 5 Centro de Referência na área das Doenças Hereditárias do Metabolismo, CHUP, 4099-001 Porto, Portugal; 6 UMIB/ICBAS/UP), Unit for Multidisplinary Research in Biomedicine, Abel Salazar Institute of Biomedical Sciences, University of Porto, 4050-313 Porto, Portugal; 7 Center for Health Technology and Services Research (CINTESIS), 4200-450 Porto, Portugal; 8 Department of Community Medicine, Information and Health Sciences (MEDCIDS), Faculty of Medicine, University of Porto, 4200-450 Porto, Portugal; 9 Faculdade de Ciências da Nutrição e Alimentação, Universidade do Porto, 4150-180 Porto, Portugal; 10 Nutrition and Metabolism, Nova Medical School, Faculdade de Ciências Médicas, Universidade Nova de Lisboa, 1169-056 Lisbon, Portugal

**Keywords:** Casein glycomacropeptide, Amino acids, Nutritional status, Phenylketonuria, Phenylalanine, Tyrosine

## Abstract

**Background:**

In phenylketonuria (PKU), modified casein glycomacropeptide supplements (CGMP-AA) are used as an alternative to the traditional phenylalanine (Phe)-free L-amino acid supplements (L-AA). However, studies focusing on the long-term nutritional status of CGMP-AA are lacking. This retrospective study evaluated the long-term impact of CGMP-AA over a mean of 29 months in 11 patients with a mean age at CGMP-AA onset of 28 years (range 15–43) [8 females; 2 hyperphenylalaninaemia (HPA), 3 mild PKU, 3 classical PKU and 3 late-diagnosed]. Outcome measures included metabolic control, anthropometry, body composition and biochemical parameters.

**Results:**

CGMP-AA, providing 66% of protein equivalent intake from protein substitute, was associated with no significant change in blood Phe with CGMP-AA compared with baseline (562 ± 289 µmol/L vs 628 ± 317 µmol/L; *p* = 0.065). In contrast, blood tyrosine significantly increased on CGMP-AA (52.0 ± 19.2 μmol/L vs 61.4 ± 23.8 μmol/L; *p* = 0.027).

**Conclusions:**

Biochemical nutritional markers remained unchanged which is an encouraging finding in adults with PKU, many of whom are unable to maintain full adherence with nutritionally fortified protein substitutes. Longitudinal, prospective studies with larger sample sizes are necessary to fully understand the metabolic impact of using CGMP-AA in PKU.

## Introduction

Phenylketonuria (PKU, OMIM # 261,600) is an inborn error of phenylalanine (Phe) metabolism caused by deficiency of phenylalanine hydroxylase [1]. PKU is successfully managed by a Phe-restricted diet supplemented with Phe-free L-amino acid supplements (L-AA) [2]. In recent years, casein glycomacropeptide (CGMP) has been prescribed as an alternative protein substitute in PKU. CGMP is a whey-based bioactive peptide derived from the cheese-making process and it is potentially valuable for human health, particularly in PKU [3].

Commercial formulations of CGMP are supplemented with rate-limiting amino acids (CGMP-AA) as methionine, leucine (Leu), lysine, arginine, tyrosine (Tyr) and tryptophan [4] to improve their suitability in patients with PKU. However, a disadvantage of CGMP-AA compared with L-AA is that it contains some residual Phe. Most formulations contain around 36 mg per 20 g protein equivalent [5].

In PKU, adherence to protein substitute is an ongoing challenge [6]. Recently, an Italian research group, using a survey to characterize the dietary habits of adult patients, showed that the intrinsic features of L-AA (e.g., palatability) are a cause of poor adherence [7]. In contrast, studies addressing the overall patient acceptability of CGMP-AA compared with their usual L-AA indicate good acceptance [8]. A sensory study suggested that CGMP-AA may enhance patients’ adherence and therefore improve health status [9].

CGMP-AA has many functional and physiological properties. It acts as a prebiotic, increases the production of short-chain fatty acids (SCFA), has anti-inflammatory properties and exerts beneficial effects on bone, creating an attractive peptide for patients with PKU [10].

Over time, PKU treatment has been refined and optimized in order to avoid the negative effects on overall health status of low and inconsistent intake of a macro- and micronutrient- supplemented L-AA [11]. However, studies addressing the long-term effect of CGMP-AA on nutritional status are lacking.

The purpose of the present study is to expand on previous data published by Pinto et al. [12] by increasing the duration of follow-up of a group of 11 patients with PKU taking CGMP-AA. In Pinto’s study, blood Phe remained unchanged whereas blood Tyr increased whilst taking 57% of CGMP-AA as protein substitute in combination with L-AA [12]. A meta-analysis performed by our group also demonstrated no significant differences between CGMP-AA and L-AA for blood Phe and Tyr control in adults with PKU [8]. However, the use of CGMP-AA in children does adversely affect blood Phe control [13–15] and so there is concern about using CGMP-AA as the sole source of protein substitute in children and pregnancy. A first case report from our group revealed no deterioration of metabolic control during pregnancy when combined with L-AA [16]. A recent longitudinal, parallel, controlled study over 12 months assessing a formulation of CGMP-AA compared with L-AA on blood Phe, Tyr, Phe/Tyr ratio, biochemical nutritional status and growth in children with PKU identified no differences for the majority of the parameters that remained within the reference ranges, although CGMP-AA only provided 75% of the total protein substitute source. A significant increase in selenium and decrease in ferritin were observed [5].

The main aim of this study was to evaluate the longitudinal impact of the use of CGMP-AA in patients with PKU.

## Materials and methods

### Study design and participants selection

We conducted a retrospective longitudinal study of patients being treated for PKU and exclusively followed-up at Centro de Genética Médica, Centro Hospitalar Universitário do Porto. Patients were given CGMP-AA as their primary nitrogen source if they had difficulties with taking L-AA or if CGMP-AA was considered a suitable alternative.

The inclusion criteria were diagnosis of PKU, absence of co-existent conditions and taking CGMP-AA as part of their protein substitute prescription. Exclusion criteria were the use of sapropterin, use of large neutral amino acids, pregnancy, lack of biochemical markers or body composition analysis.

All patients followed a low-Phe diet, avoiding high protein foods, and supplemented with L-AA and special low protein foods. Phe intake was controlled using a Phe exchange system (1 exchange = 20 mg of Phe).

The present study included data on 11 patients from a previous study reported by Pinto et al. [12] but an extended follow-up period of 2.9 years if patients remained on CGMP-AA.

Data was collected between May 2013 and December 2018, whereas the previous study by Pinto et al. [12] retrieved data until February 2016. The first annual nutritional status evaluation (ANSE) was performed when taking L-AA and the last ANSE with the addition of CGMP-AA. The baseline assessment was conducted for a mean of 6 months before CGMP-AA commencement when patients were taking L-AA only as their primary nitrogen source. The last assessment was carried out when CGMP-AA had been given for a mean of 29 months. CGMP-AA either fully or partially replaced L-AA; CGMP-AA contribution to the total protein substitute intake was: 100%, *n* = 4, 50% to < 100%, *n* = 4, < 50%, *n* = 3. This was according to patient’s protein substitute preference or by the nutritionist’s prescription after assessing metabolic control, nutritional status, nutritional intake, anthropometry and body composition.

The PKU classification was based on the Portuguese guidelines as follows: hyperphenylalaninaemia (HPA) [blood Phe < 360 µmol/L (6 mg/dL)]; mild PKU [blood Phe ≥ 360 µmol/L and ≤ 1200 µmol/L (≥ 6 mg/dL and ≤ 20 mg/dL)] and classical PKU [blood Phe > 1200 µmol/L (> 20 mg/dL)] [17].

Blood Phe and Tyr control was also evaluated over 2-time intervals as follows: i) from May 2013 until CGMP-AA introduction (13 ± 5 months) and ii) from CGMP-AA introduction until the last ANSE taking CGMP-AA (29 ± 16 months). The median number of blood Phe measurements while patients were taking L-AA was 11 (7–16) and with CGMP-AA was 40 (21–71).

**Fig. 1:**
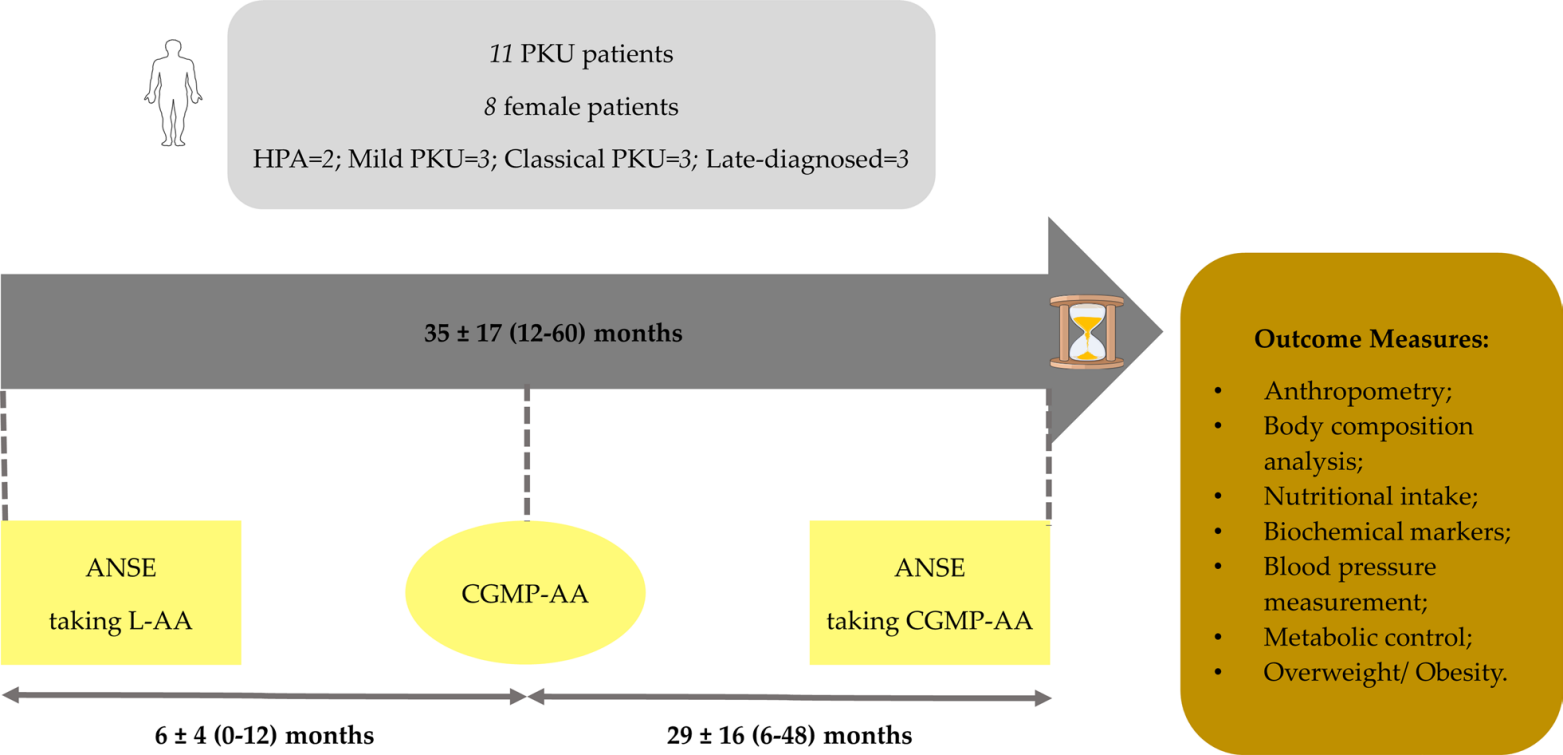
Study design. ANSE, annual nutritional status evaluation; CGMP-AA, casein glycomacropeptide supplements; HPA, hyperphenylalaninaemia; L-AA, phenylalanine-free L-amino acid supplements; PKU, phenylketonuria

The study design is presented in Fig. 1.

### Data collection and outcomes measured

The following parameters were collected from patients’ records by trained research nutritionists (M.J.P. and A.P.):

### Anthropometry

Height (cm) was measured with light clothes, using a stadiometer (SECA GmbH & CO., Hamburg, Germany) (measured to the nearest millimetre) and weight (kg) was assessed with a mechanical weighing scale (SECA GmbH & CO., Hamburg, Germany) (measured to the nearest 100 g). Waist circumference (WC) (cm) was measured in the standing position, midway between the lower rib margin and the iliac crest, at the end of a normal exhalation, to the nearest millimetre and using a non-extensive metric tape. Anthropometric measures were performed by trained nutritionists (M.F.A. and J.C.R.).

### Body composition analysis

Body composition was performed in the fasted state using a single-frequency (50 Hz) bioelectrical impedance analyzer, Akern, Quantum/S (RJL systems, Florence, Italy) according to described standards and measurement conditions. Total fat mass, percentage of body fat mass, percentage of lean mass and phase angle were assessed in the programme BodyGram™ version 1.3 (Akern Bioresearch, Florence, Italy) which uses validated prediction equations [18]. The measures were carried out by trained nutritionists (M.F.A. and J.C.R.).

### Nutritional intake

Total protein intake, natural protein intake (g/kg/day), protein equivalent from the protein substitute (g/kg/day and g/day), Phe intake (mg/day) from both natural foods and CGMP-AA, Tyr (g/day) and Leu (g/day) intake from the protein substitutes were calculated using a 24-h food recall. Dietary assessments were performed by M.F.A. and J.C.R..

### Biochemical markers

Blood samples for biochemical analysis were taken after an overnight fast. Uric acid, glucose, creatinine, urea, glycated haemoglobin (HbA1c), lipid panel [total cholesterol, triglycerides, high-density lipoprotein (HDL)-cholesterol, low-density lipoprotein (LDL)-cholesterol, very low-density lipoproteins (VLDL)-cholesterol, apolipoprotein A1, apolipoprotein B], iron, transferrin, ferritin, albumin, homocysteine, prealbumin, C-reactive protein, insulin, calcium, phosphorus, selenium, zinc, vitamin B12, vitamin D and folic acid were determined. Blood urea nitrogen (BUN) was calculated from urea and homeostatic model of insulin resistance (HOMA-IR) was calculated as follows: HOMA-IR = fasting plasma glucose (mg/dL) × fasting serum insulin (μU/mL)/405 [19].

### Blood pressure

A Critikon Dinamap™ vital signs monitor 8100 was used to measure resting systolic and diastolic blood pressure and heart rate with individuals seated for at least 5 min, according to standard techniques.

### Metabolic control

Blood Phe and Tyr were measured by fasting blood spots and analysed by tandem mass spectrometry. Patients or Caregivers were trained to perform routine blood spots. Good metabolic control was defined as median blood Phe level within 120–360 µmol/L (2–6 mg/dL) ≤ 12 years or 120–480 µmol/L (2–8 mg/dL) > 12 years of age, according to the Portuguese criteria [20]. The percentage of median blood Phe within the target range was also calculated.

### Overweight/obesity

Body mass index (BMI) was calculated as the ratio of weight (kg) and height (m^2^) and classified according to the World Health Organisation (WHO) criteria. Overweight and obesity were defined when BMI was between 25.0 and 29.9 kg/m^2^ or was ≥ 30.0 kg/m^2^, respectively [21]. The Anthro Plus® programme version 1.0.4 was used to calculate the BMI z-scores for individuals under 19 years. Overweight and obesity were identified when the BMI z-score was between 1 and 2 standard deviations (S.D.) or above 2 S.D., respectively [22].

### Statistical analysis

All statistical analyses were performed with SPSS® version 26.0 for Mac (IBM Company, Chicago, IL, USA). Normal distribution was checked using Shapiro–Wilk test. Categorical variables are expressed as percentage and continuous variables as mean ± S.D. or median (P25–P75) where appropriate. Paired t-test and Wilcoxon signed ranks test  were used to analyse the differences when normal distribution or non-normal was found, respectively. The McNemar test was used to determine if there are differences on a dichotomous dependent variable between 2 related groups. Significance was set at the level of *p* value less than 0.05.

## Results

### Characteristics of patients with PKU

Table 1 summarizes the main features of the 11 patients included in this study (8 females and 3 males). The mean age at CGMP-AA onset was 28 years (range 15 to 43) (1 patient was < 18 years: 15 years, *n* = 1). In this cohort of patients, 2 of 11 had HPA, 3 mild PKU, 3 classical PKU and 3 were late-diagnosed. The mean length of time on CGMP-AA was 29 months (range 6 to 48).

**Table 1:** Characteristics of patients with PKU included in the study

Patient ID	Blood Phe levels at NBS (µmol/L)	Genotype		PKU classification	Age at ANSE under L-AA (years)	Age at CGMP-AA start (years)	Number of months taking CGMP-AA	Contribution of CGMP-AA to the total protein substitute intake at last assessment (%)
1	240	L249F	A300S	HPA	17	18	6	100
2	N/A	I65T	R270K	Late-diagnosed	42	43	47	60
3	720	I65T	I65T	Mild PKU	26	27	27	23
4	N/A	IVS10-11G>A	V388M	Late-diagnosed	40	41	42	60
5	1260	IVS11+5G>A	165T	Classical PKU	25	26	38	100
6	N/A	R158Q	R252W	Late-diagnosed	37	38	7	27
7	780	I65T	IVS10-11G>A	Mild PKU	18	20	20	28
8	2580	IVS10-11G>A	IVS10-11G>A	Classical PKU	24	27	48	100
9	1260	P281L	P281L	Classical PKU	29	31	16	52
10	420	R261Q	E390G	HPA	13	15	44	100
11	840	IVS10-11G>A	R270K	Mild PKU	21	23	21	75

ANSE, annual nutritional status evaluation; CGMP-AA, casein glycomacropeptide supplements; HPA, hyperphenylalaninaemia; ID, identification; L-AA, phenylalanine-free L-amino acid supplements; N/A, not available; NBS, newborn screening; Phe, phenylalanine; PKU, phenylketonuria

### Nutritional intake

Table 2 shows the type of protein substitute used in each assessment. The L-AA formulations prescribed were mainly powders and liquids. Subjects usually took more than one type of L-AA. The majority of patients (*n* = 9; 82%) were treated with the same formulation of CGMP-AA, Glytactin BetterMilk® (Cambrooke, USA). Five of 11 patients took CGMP-AA for less than 2 years. The reasons why patients stopped taking CGMP-AA were poor dietary adherence (*n* = 1), temporary loss of follow-up (*n* = 1), sapropterin therapy (*n* = 2) and pregnancy (*n* = 1).

**Table 2:** Type of protein substitute used in each assessment

Forms	L-AAt	CGMP-AAt	
		L-AA	CGMP-AA
Powders	PKU 3 Advanta® (Nutricia) (*n* = 4)	PKU 3 Advanta® (Nutricia) (*n* = 2)	Glytactin BetterMilk® (Cambrooke) (*n* = 9)
	PhenylAde® (Taranis) (*n* = 2)		
	PKU 2 Secunda® (Nutricia) (*n* = 1)		
	PKU Anamix Junior® (Nutricia) (*n* = 1)		
	Phlexy 10 Drink Mix® (Nutricia) (*n* = 1)		
Liquids	PKU Cooler 10, 15 and 20® (Vitaflo) (*n* = 9) XPhe Jump 10® (MetaX) (*n* = 1)	PKU Cooler 10, 15 and 20® (Vitaflo) (*n* = 5) PKU Lophlex LQ 10® (Nutricia) (*n* = 1)	Glytactin RTD 15® (Cambrooke) (*n* = 2) Glytactin RTD 10® (Cambrooke) (*n* = 3)
Bars	PhenylAde Amino Acid Bar® (Taranis) (*n* = 1)		

CGMP-AA, casein glycomacropeptide supplements; L-AA, phenylalanine-free L-amino acid supplements; PKU, phenylketonuria; RTD, ready to drink; L-AAt, type of protein substitute when taking L-AA; CGMP-AAt, type of protein substitute when taking CGMP-AA

At the last ANSE, CGMP-AA contributed a mean of 66 ± 31% (range 23 to 100) to the total protein substitute intake. The mean Phe provided by CGMP-AA was 44 mg/day (range 23 to 73).

Table 3 describes metabolic control, nutritional intake, anthropometry and body composition of the participants when taking L-AA compared with CGMP-AA. The total amount of protein equivalent from protein substitute remained unchanged [(0.86 ± 0.24 g/kg/day vs 0.74 ± 0.23 g/kg/day; *p* = 0.126) and (50.8 ± 16.3 g/day vs 44.6 ± 12.8 g/day; *p* = 0.118)]. The intake of Tyr was not affected with CGMP-AA (5.18 ± 1.77 g/day vs 4.22 ± 1.59 g/day; *p* = 0.145). Natural protein and Phe ingestion stratified according to the percentage of CGMP-AA intake of the 11 patients with PKU taking L-AA vs CGMP-AA is shown in Additional file 1: Table S1.

**Table 3:** Overall metabolic control, nutritional intake, anthropometry and body composition of the 11 patients with PKU taking L-AA versus CGMP-AA

Profile	*n*	T_L-AA_	T_CGMP-AA_	*p* value
Metabolic control				
Median blood Phe (µmol/L)	11	562 ± 289	628 ± 317	0.065
Median blood Tyr (μmol/L)	11	52.0 ± 19.2	61.4 ± 23.8	**0.027**
Median blood Phe/Tyr ratio	11	15.1 ± 10.8	12.0 ± 8.8	0.379
Nutritional intake				
Natural protein intake (g/kg/day)	11	0.41 (0.26–0.62)	0.34 (0.21–0.69)	0.657
Protein substitute (g/kg/day)	11	0.86 ± 0.24	0.74 ± 0.23	0.126
Protein substitute (g/day)	11	50.8 ± 16.3	44.6 ± 12.8	0.118
Phe intake (mg/day)	11	885 (751–1787)	978 (658–1370)	0.721
Phe intake from protein substitute (mg/day)	11	0	43.5 ± 17.4	–
Tyr intake from protein substitute (g/day)	11	5.18 ± 1.77	4.22 ± 1.59	0.145
Leu intake from protein substitute (g/day)	11	5.85 (4.49–7.61)	7.97 (6.85–8.78)	0.075
Protein (%)	11	13.8 ± 2.25	13.6 ± 2.25	0.696
Fat (%)	11	26.9 ± 5.0	27.6 ± 4.2	0.503
CHO (%)	11	57.0 (52.1–60.1)	58.5 (55.8–60.2)	0.248
Energy (kcal/day)	11	2277 ± 551	2238 ± 491	0.793
Anthropometry and body composition				
Weight (kg)	11	60.4 ± 15.2	63.4 ± 13.2	0.064
Height (cm)	11	158.6 ± 6.4	160.6 ± 9.8	0.341
WC (cm)	11	85.1 ± 15.2	86.2 ± 14.3	0.536
BMI (kg/m^2^) *	9	24.3 ± 6.2	25.1 ± 5.6	0.095
Body fat (kg)	9	17.3 ± 13.5	19.5 ± 12.3	0.056
Body fat (%)	9	25.5 ± 16.1	28.9 ± 13.4	0.126
Lean mass (%)	9	74.5 ± 16.1	71.1 ± 13.4	0.126
Phase angle (°)	9	6.8 ± 0.7	6.8 ± 0.6	0.880

BMI, body mass index; CHO, carbohydrate; CGMP-AA, casein glycomacropeptide supplements; L-AA, phenylalanine-free L-amino acid supplements; Leu, leucine; Phe, phenylalanine; PKU, phenylketonuria; Tyr, tyrosine; T_L-AA_, annual nutritional status evaluation under L-AA; T_CGMP-AA_, last annual nutritional status evaluation under CGMP-AA; WC, waist circumference. Data are presented as mean ± S.D. (*n*) or median (P25–P75) (*n*). Paired t-test and Wilcoxon test were performed to identify differences when normal distribution or non-normal was found, respectively. Significance was set at the level of *p* value less than 0.05 and highlighted in bold. * Mean BMI was only calculated for adults

### Anthropometry and body composition analysis

Table 3 describes parameters of anthropometry and body composition of all patients taking L-AA compared with CGMP-AA. Patients on CGMP-AA had a tendency for increased body weight (60.4 ± 15.2 kg vs 63.4 ± 13.2 kg; *p* = 0.064) and total body fat (17.3 ± 13.5 kg vs 19.5 ± 12.3 kg; *p* = 0.056) when compared to baseline with L-AA. However, the overall percentage of overweight and obesity in patients taking L-AA (46%) vs CGMP-AA (46%) remained unchanged (*p* = 1.000).

### Biochemical markers and blood pressure

There were no differences in the biochemical and blood pressure data (Table 4).

**Table 4:** Blood pressure and biochemical data of patients at baseline compared with last assessment

Profile	*n*	T_L-AA_	T_CGMP-AA_	*p* value
Systolic blood pressure (mmHg)	11	112 ± 13	112 ± 11	1.000
Diastolic blood pressure (mmHg)	11	60 ± 15	63 ± 12	0.581
Heart rate (bpm)	11	73 ± 9	72 ± 8	0.607
Uric acid (mg/dL)	11	4.0 ± 0.8	4.0 ± 1.2	0.847
Glucose (mg/dL)	11	78.2 ± 6.8	75.6 ± 5.3	0.190
Creatinine (mg/dL)	11	0.7 ± 0.1	0.7 ± 0.1	0.676
Urea (mg/dL)	11	20.5 ± 7.6	23.2 ± 6.7	0.262
BUN (mg/dL)	11	1.68 ± 0.63	1.90 ± 0.55	0.268
HbA1c (%)	10	5.1 ± 0.4	5.0 ± 0.3	0.107
Total cholesterol (mg/dL)	11	165 ± 37	156 ± 34	0.349
HDL-C (mg/dL)	11	55 ± 14	51 ± 11	0.194
LDL-C (mg/dL)	11	93 ± 32	85 ± 32	0.265
VLDL-C (mg/dL)	11	17 ± 6	20 ± 10	0.121
Triglycerides (mg/dL)	11	84 ± 27	99 ± 50	0.140
ApoA1 (mg/dL)	10	153 ± 25	145 ± 31	0.187
ApoB (mg/dL)	10	82 ± 22	80 ± 22	0.612
Iron (μg/dL)	11	115 (85–135)	88 (48–144)	0.266
Transferrin (mg/dL)	11	271 ± 43	263 ± 44	0.554
Ferritin (ng/dL)	11	65 ± 35	52 ± 26	0.132
Albumin (g/dL)	11	4.69 ± 0.33	4.57 ± 0.17	0.112
Homocysteine (μmol/L)	8	8.44 ± 1.51	8.21 ± 1.32	0.820
Prealbumin (mg/dL)	11	240 (224–278)	272 (200–293)	0.575
CRP (mg/dL)	10	0.89 (0.72–2.46)	1.28 (0.77–2.92)	0.799
Insulin (μU/mL)	8	7.8 (7.3–13.6)	9.8 (6.5–18.3)	0.161
HOMA-IR	8	1.5 (1.4–2.5)	1.8 (1.2–3.5)	0.327
Calcium (mmol/L)	11	2.37 ± 0.12	2.33 ± 0.11	0.164
Phosphorus (mmol/L)	11	1.07 ± 0.24	0.98 ± 0.14	0.221
Selenium (μmol/L)	6	0.70 ± 0.30	0.96 ± 0.31	0.257
Zinc (μmol/L)	11	12.5 (9.7–17.4)	11.3 (10.4–12.4)	0.155
Vit. B12 (pg/L)	11	484 (413–734)	459 (301–1043)	0.722
Vit. D (nmol/L)	11	77 ± 26	70 ± 27	0.153
Folic acid (ng/mL)	9	12.9 ± 3.8	12.9 ± 5.3	0.996

ApoA1, apolipoprotein A1; ApoB, apolipoprotein B; BUN, blood urea nitrogen; CGMP-AA, casein glycomacropeptide supplements; C, cholesterol; CRP, c-reactive protein; HbA1c, glycated haemoglobin; HDL-C, high-density lipoprotein-cholesterol; HOMA-IR, homeostatic model of insulin resistance; L-AA, phenylalanine-free L-amino acid supplements; LDL-C, low-density lipoprotein-cholesterol; Phe, phenylalanine; PKU, phenylketonuria; Tyr, tyrosine; T_L-AA_, nutritional status evaluation under L-AA; T_CGMP-AA_, last annual nutritional status evaluation under CGMP-AA; Vit, vitamin; VLDL-C, very low-density lipoprotein-cholesterol. Data are presented as mean ± S.D. (*n*) or median (P25–P75) (*n*). Paired t-test and Wilcoxon test were performed to identify differences when normal distribution or non-normal was found, respectively. Significance was set at the level of *p* value less than 0.05

### Metabolic control

Table 3 describes metabolic control. Blood Phe concentrations were similar between baseline and CGMP-AA (562 ± 289 µmol/L vs 628 ± 317 µmol/L; *p* = 0.065). The percentage of patients with median blood Phe within target range did not change (36% vs 36%, *p* = 1.000). In the subgroup of patients with 100% CGMP-AA (*n* = 4), half of the patients had good metabolic control and the other half did not.

Blood Tyr significantly increased with CGMP-AA (52.0 ± 19.2 μmol/L vs 61.4 ± 23.8 μmol/L; *p* = 0.027). Metabolic control stratified according to the percentage of CGMP-AA intake of the 11 patients with PKU taking L-AA vs CGMP-AA is shown in Additional file 1: Table S2.

**Fig. 2:**
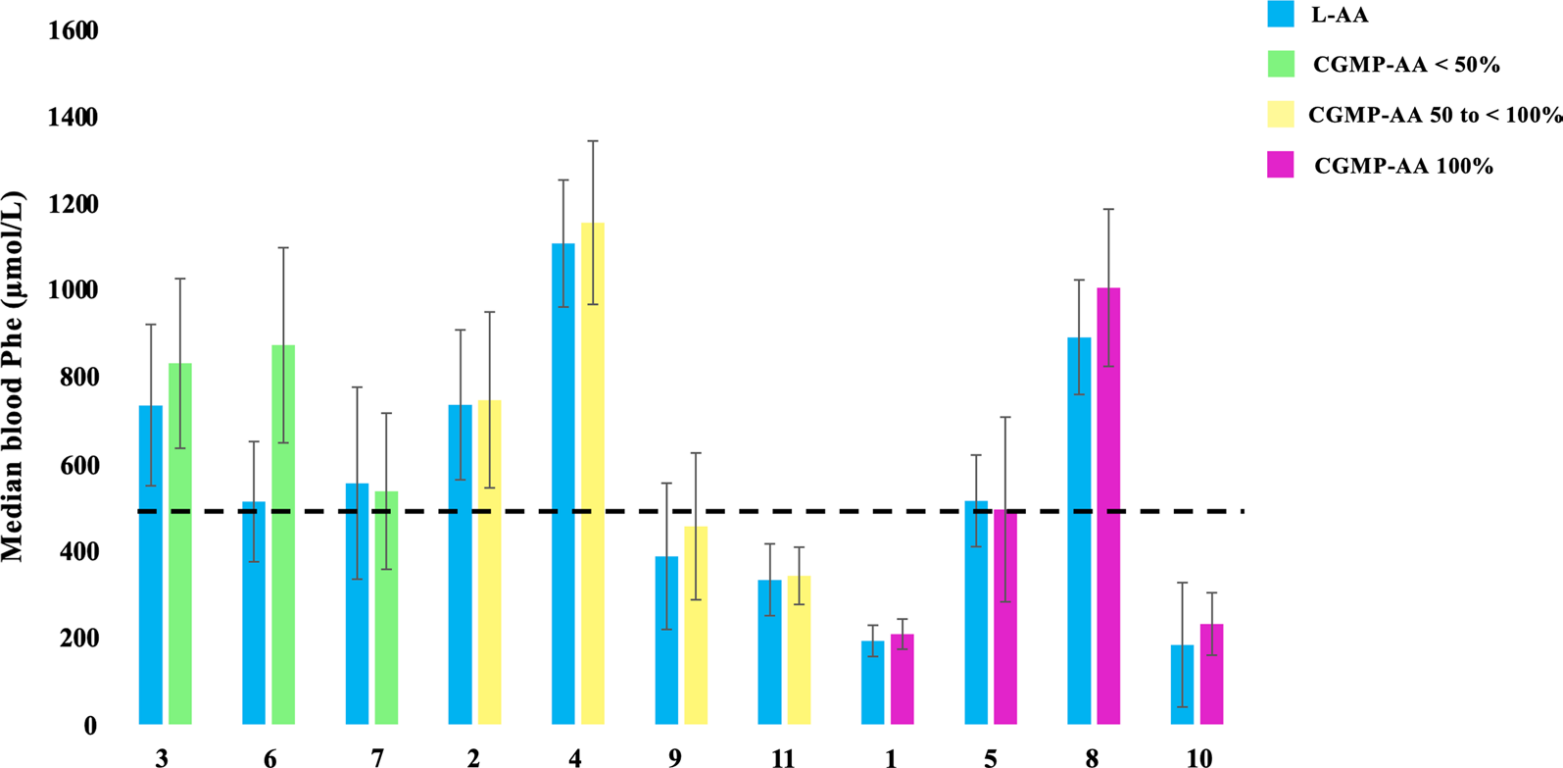
Median blood Phe levels of 11 patients with PKU taking L-AA versus CGMP-AA (last ANSE) with different percentages of contribution to the total protein substitute. CGMP-AA, casein glycomacropeptide supplements; L-AA, phenylalanine-free L-amino acid supplements; Phe, phenylalanine. Dashed line: target level of 480 µmol/L > 12 years. The number below each pair of bars represents Patient ID

**Fig. 3:**
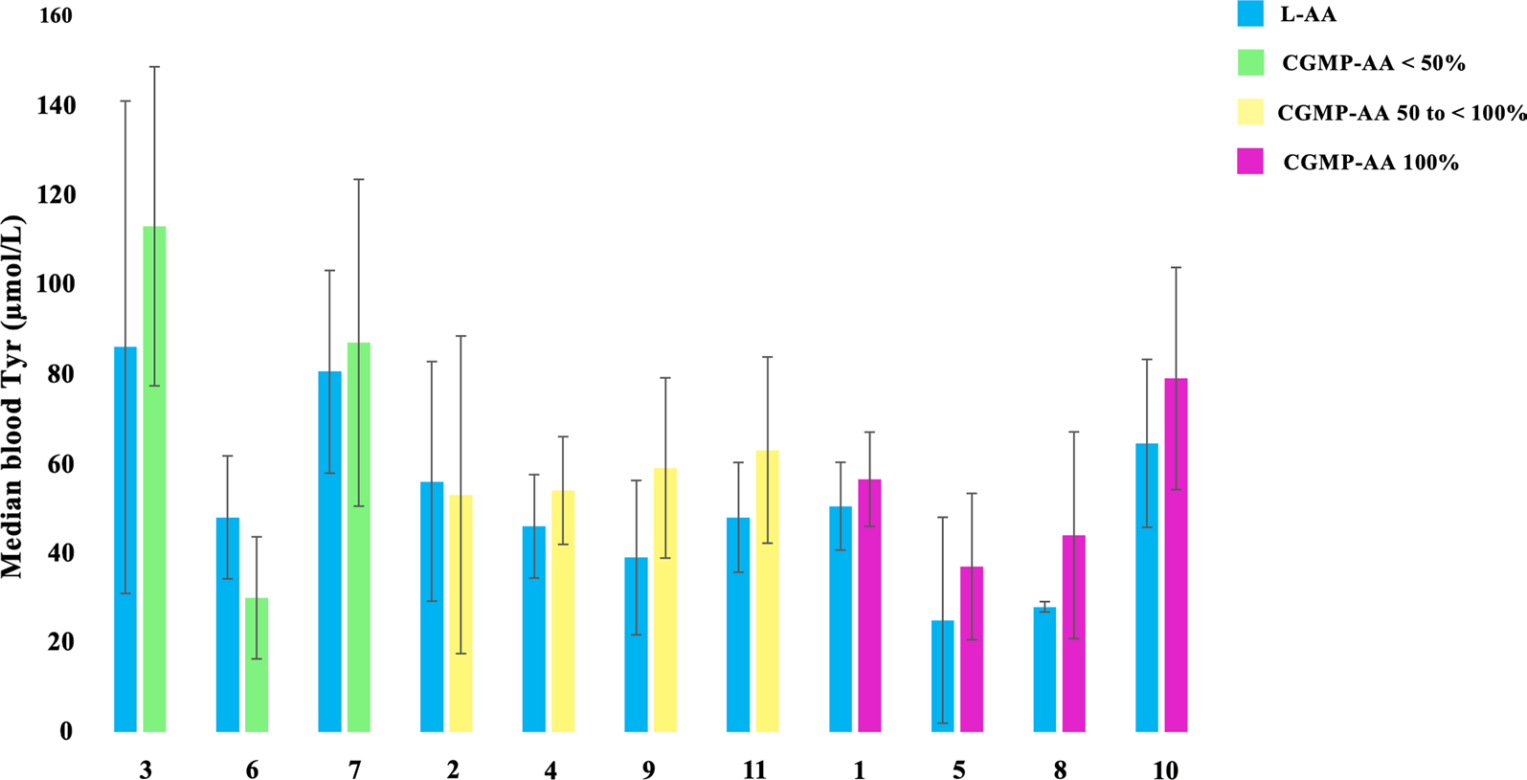
Median blood Tyr levels of 11 patients with PKU taking L-AA versus CGMP-AA (last ANSE) with different percentages of contribution to the total protein substitute. CGMP-AA, casein glycomacropeptide supplements; L-AA, phenylalanine-free L-amino acid supplements; Tyr, tyrosine. The number below each pair of bars represents Patient ID

Figures 2 and 3 show the metabolic control of Phe and Tyr control between baseline and the last assessment.

## Discussion

This PKU study was the continuation of the work performed by Pinto et al. [12] and it is the only report describing the nutritional status of adults taking CGMP-AA for a mean period of 2.4 years. CGMP-AA provided a mean 66% of the total protein substitute source (only 4 patients took 100% of protein substitute requirement as CGMP-AA), which was a small increase compared with the 57% from our previous report [12]. Only 6 of 11 patients took CGMP-AA for over 2 years.

There were no changes in blood Phe levels in this older cohort of patients with PKU, which is in line with the previous findings with CGMP-AA studies [12], although patients ≥ 12 years of age maintained higher upper target blood Phe levels than children. In addition, half of our cohort had mild forms of PKU with a high Phe tolerance and so any extra Phe provided by CGMP-AA might not have affected the blood Phe levels [23]. Also, CGMP-AA only contributed partially to the total protein substitute intake [24]. Finally, this was a small cohort of patients and the study was not powered to find a statistical change based on such small numbers.

Interestingly, an increase in blood Tyr was observed despite no difference in intake. The putative explanation for this finding can be related to better adherence with the protein substitute. Although L-AA contains higher amounts of Tyr compared to CGMP-AA, its bioavailability may be compromised due to lower solubility properties [25]. There is a suggestion that gut microbiota may contribute to less bioavailability of Tyr from L-AA [26], leading to lower blood Tyr levels. Considering diet is one of the main factors determining the gut microbiota which has been shown to interact with host metabolism [27], it is important to explore how the synthetic diet in PKU influences the configuration of the microbial community in the gut. It is possible that CGMP-AA also influences its composition. Theoretically, a diet poor or rich in certain nutrients may trigger an intestinal dysbiosis with systemic repercussions, such as obesity, diabetes, cancer, among others [28]. Studies sought to identify the effects of PKU diet on the microbiota are scarce, especially with CGMP-AA [26, 29].

In contrast to the Pinto et al. [12] study, HbA1c did not change in patients taking CGMP-AA. Even though, L-AA seems to lower glucose levels when compared to intact protein [30], further studies are needed to understand how chronic administration of protein substitute may influence glucose metabolism.

Biochemical biomarkers remained unchanged. BUN, a serum byproduct of protein metabolism that can be affected by dietary protein intake was similar to baseline on L-AA. This was comparable to previous reports [13, 31, 32] but also contradicts studies that found significantly lower levels with CGMP-AA [33, 34].

There was no impact on body composition and there was a trend for increased weight. The specific brand of CGMP-AA used by the majority of patients was higher in energy compared with the L-AA given. There is suggestion that CGMP-AA increases satiety and a similar energy intake between baseline and the last assessment was observed [35]. However, any change in weight should be interpreted carefully. In this study, patient numbers were small and data on physical activity was not collected. Daly et al. studied a group of children taking CGMP-AA vs L-AA over 12 months. They found no differences between CGMP-AA and L-AA for anthropometry at each of the measured time points but within the CGMP-AA group, weight and BMI z-scores increased significantly between baseline to 12 months [5].

The results from our study are very encouraging about the use of CGMP-AA in adult patients with PKU. It is well established that dietary non-adherence increases with age, commonly reflecting poor tolerance of the protein substitute [6]. The restrictive nature of the PKU diet as well as inadequate adherence with the fortified protein substitute is likely to cause sub-optimal nutritional intake and increase the risk of clinical and biochemical nutritional deficiencies [36]. In this study, CGMP-AA enabled many adult patients to successfully remain on dietary treatment. Only 1 of 5 patients stopped taking CGMP-AA due to poor adherence.

Our findings do have several limitations. Although we collected data over 6 years, the sample size is still small and only 55% (6 out 11 patients) of the patient cohort had > 2 years follow-up whilst taking CGMP-AA. Also, not all the patients replaced their full prescription of L-AA intake with CGMP-AA. In addition, the severity of disorder varied widely, with 5 patients having a milder phenotype, with increased Phe tolerance [23], minimising the impact of CGMP-AA on blood Phe levels. Also, patients’ blood Phe control at the start of the study was not optimal. This study had a retrospective design and dietary adherence is known to decrease with increasing age. We had no specific marker of adherence with L-AA or CGMP-AA, although we examined many biochemical and nutritional parameters. Dietary intake was collected by a 24-h food recall method, and like other dietary assessment methods it is associated with disadvantages.

## Conclusions

With this long-term work we can clearly state that the metabolic control and the biochemical nutritional status of patients with PKU did not deteriorate with time, but only 6 patients remained on CGMP-AA > 2 years duration. In addition, parameters of body composition and the percentage of overweight and obesity did not significantly increase while taking CGMP-AA for a longer follow-up period.

This work suggests that CGMP-AA is safe and does not affect nutritional status even though it would be useful to have more studies replicating these results with a higher sample of patients. So far, the only difference observed to traditional L-AA is improved palatability which may contribute to better long-term adherence with protein substitute.

## Supplementary Information

**Additional file 1.** Supplementary data.

## Data Availability

The datasets during and/or analysed during the current study available from the corresponding author on reasonable request.

## Abbreviations

ANSE: Annual nutritional status evaluation
ApoA1: Apolipoprotein A1
ApoB: Apolipoprotein B
BMI: Body mass index
BUN: Blood urea nitrogen
CGMP: Casein glycomacropeptide
CGMP-AA: Casein glycomacropeptide supplements
C: Cholesterol
CHO: Carbohydrate
CRP: C-reactive protein
HbA1c: Glycated haemoglobin
HDL-C: High-density lipoprotein-cholesterol
HOMA-IR: Homeostatic model of insulin resistance
HPA: Hyperphenylalaninaemia
L-AA: Phenylalanine (Phe)-free L-amino acid supplements
Leu: Leucine
LDL-C: Low-density lipoprotein-cholesterol
NBS: Newborn screening
Phe: Phenylalanine
PKU: Phenylketonuria
RTD: Ready to drink
SCFA: Short-chain fatty acids
SD: Standard deviation
T_CGMP-AA_: Last annual nutritional status evaluation under CGMP-AA
T_L-AA_: Nutritional status evaluation under L-AA
Tyr: Tyrosine
VLDL-C: Very low-density lipoprotein-cholesterol
Vit: Vitamin
WC: Waist circumference
WHO: World Health Organisation
