# Supplementary material for: Continuous use of glycomacropeptide in the nutritional management of patients with phenylketonuria: a clinical perspective
Source: Orphanet J Rare Dis. 2021 Feb 13;16:84. doi: 10.1186/s13023-021-01721-8 (PMC7881530; doi:10.1186/s13023-021-01721-8)
Supplement: Supplementary file 1 — Additional file 1. Supplementary data. [file 13023_2021_1721_MOESM1_ESM.docx]

**Supplementary Table 1.** Natural protein and Phe ingestion stratified according to the percentage of CGMP-AA intake of the 11 patients with PKU taking L-AA vs CGMP-AA.

| **Profile** | **NP (g/d) T_L-AA_** | **NP (g/d) T_CGMP-AA_** | ***p*-value** | **Phe (mg/d) T_L-AA_** | **Phe (mg/d) T_CGMP-AA_** | ***p*-value** |
| --- | --- | --- | --- | --- | --- | --- |
| **Patients with CGMP < 50% (*n*=3)** |  |  |  |  |  |  |
| 3 | 12.5 | 10.0 | 0.555 | 513 | 425 | 0.926 |
| 6 | 19.0 | 11.0 |  | 751 | 448 |  |
| 7 | 13.5 | 17.0 |  | 536 | 868 |  |
| **Patients with CGMP 50 to < 100% (*n*=4)** |  |  |  |  |  |  |
| 2 | 25.7 | 16.2 | 0.180 | 796 | 817 | 0.226 |
| 4 | 18.5 | 20.7 |  | 885 | 1014 |  |
| 9 | 23.8 | 14.9 |  | 1787 | 658 |  |
| 11 | 30.9 | 27.9 |  | 2641 | 1308 |  |
| **Patients with CGMP 100% (*n*=4)** |  |  |  |  |  |  |
| 1 | 42.5 | 62.6 | 0.199 | 3002 | 3002 | 0.109 |
| 5 | 20.4 | 20.6 |  | 876 | 978 |  |
| 8 | 29.4 | 30.0 |  | 934 | 1370 |  |
| 10 | 44.6 | 81.5 |  | 1241 | 4009 |  |

CGMP-AA: casein glycomacropeptide supplements; L-AA: phenylalanine-free L-amino acid supplements; NP: natural protein; Phe: phenylalanine; PKU: phenylketonuria; T_L-AA_: annual nutritional status evaluation under L-AA; T_CGMP-AA_: last annual nutritional status evaluation under CGMP-AA Data are presented as raw values. Paired t-test and Wilcoxon test were performed to identify differences when normal distribution or non-normal was found, respectively. Significance was set at the level of *p*-value less than 0.05 and highlighted in bold.

**Supplementary Table 2.** Metabolic control stratified according to the percentage of CGMP-AA intake of the 11 patients with PKU taking L-AA vs CGMP-AA.

| **Profile** | **Median blood Phe (µmol/L) T_L-AA_** | **Median blood Phe (µmol/L) T_CGMP-AA_** | ***p*-value** | **Median blood Tyr (µmol/L) T_L-AA_** | **Median blood Tyr (µmol/L) T_CGMP-AA_** | ***p*-value** |
| --- | --- | --- | --- | --- | --- | --- |
| **Patients with CGMP < 50% (*n*=3)** |  |  |  |  |  |  |
| 3 | 738 | 834 | 0.322 | 86 | 113 | 0.730 |
| 6 | 516 | 876 |  | 48 | 30 |  |
| 7 | 558 | 540 |  | 81 | 87 |  |
| **Patients with CGMP 50 to < 100% (*n*=4)** |  |  |  |  |  |  |
| 2 | 739 | 750 | 0.101 | 56 | 53 | 0.138 |
| 4 | 1110 | 1158 |  | 46 | 54 |  |
| 9 | 390 | 459 |  | 39 | 59 |  |
| 11 | 336 | 345 |  | 48 | 63 |  |
| **Patients with CGMP 100% (*n*=4)** |  |  |  |  |  |  |
| 1 | 195 | 211 | 0.259 | 51 | 57 | **0.012** |
| 5 | 518 | 498 |  | 25 | 37 |  |
| 8 | 894 | 1008 |  | 28 | 44 |  |
| 10 | 186 | 234 |  | 65 | 79 |  |

CGMP-AA: casein glycomacropeptide supplements; L-AA: phenylalanine-free L-amino acid supplements; Phe: phenylalanine; PKU: phenylketonuria; Tyr: tyrosine; T_L-AA_: annual nutritional status evaluation under L-AA; T_CGMP-AA_: last annual nutritional status evaluation under CGMP-AA. Data are presented as raw values. Paired t-test was performed to identify differences. Significance was set at the level of *p*-value less than 0.05 and highlighted in bold.
